# Lymph node ratio and overall survival in patients with colon cancer: a prospective multicentre cohort study

**DOI:** 10.1038/s41598-026-50360-1

**Published:** 2026-04-27

**Authors:** Marcin Ekman, Elzbieta Jodłowska-Siewert, Marcin Urbanowicz, Maciej Berut, Alan Grabowski, Bartosz Kapturkiewicz, Michał Nycz, Michał Putko, Arkadiusz Szarmach, Wojciech Dudzic, Tomasz Jastrzębski

**Affiliations:** 1 Department of Surgical Oncology, Transplant Surgery and General Surgery, Medical University of Gdansk, Gdansk, Poland; 2 Division of Biostatistics and Health Data Science, University of Minnesota, Minnesota, USA; 3 1Nalecz Institute of Biocybernetics and Biomedical Engineering, Polish Academy of Sciences, Warsaw, Poland; 4 Department of General Surgery with Subdivision of Oncological Surgery, District Health Center in Brzeziny, Brzeziny, Poland; 5 Department of Surgery, Clinic of Surgical Oncology and Neuroendocrine Tumors, Maria Sklodowska-Curie National Research Institute of Oncology, Warsaw, Poland; 6 First Department of Oncological Surgery of Lower Silesian Oncology Center, Wroclaw, Poland; 7 Department of General, Colorectal and Polytrauma Surgery, Faculty of Health Sciences in Katowice, Medical University of Silesia, Katowice, Poland; 8 Department of Imaging Diagnostics, Specialist Hospital in Koscierzyna Ltd., Koscierzyna, Poland; 9 Hernia Center, Luxmed Hospital in Gdansk, Gdansk, Poland; 10 Department of Vascular Surgery, St. Vincent a’Paulo Hospital, Gdynia, Poland; 11 Division of Gynecology and Obstetrics, Medical University of Gdansk, Gdansk, Poland; 12 University Clinical Centre in Gdansk, Gdansk, Poland

**Keywords:** Gastrointestinal cancer, Surgical oncology

## Abstract

Colorectal cancer (CRC) is a major health concern globally, with lymph node metastasis being a key factor in its spread. The lymph node ratio (LNR), defined as the ratio of pathologically positive lymph nodes to the total number of examined nodes, has been proposed as a prognostic marker in CRC. However, data from prospective cohort studies remain limited. This study aimed to evaluate the association between LNR and overall survival (OS) in patients with colon cancer in a prospective multicentre cohort. A prospective cohort study was conducted across seven Polish surgical centers enrolling 445 patients with primary colon cancer. The study collected survival data until September 30, 2022, focusing on patients aged 18–75 years eligible for radical surgery. Preoperative assessments included demographic information, Body Mass Index (BMI) and histopathological evaluation according to AJCC 8th edition. Surgical complications were graded using the Clavien-Dindo scale. The association between OS and LNR was analysed using a multivariable Cox proportional hazards model adjusting for selected clinical and surgical covariates. In the multivariable Cox model, LNR, BMI, and grade III or higher postoperative complications were independently associated with overall survival. For LNR, a 0.1-unit increase was associated with a 26% increase in the hazard of death (hazard ratio 1.26, 95% CI 1.07–1.48; *p* = 0.006). In this prospective multicentre cohort, lymph node ratio was associated with overall survival in patients with colon cancer, alongside body mass index and the severity of postoperative complications. These findings suggest that LNR may represent a clinically relevant prognostic marker and warrant further investigation in studies specifically designed to compare its prognostic performance with established nodal staging systems.

**Supplementary Information:**

The online version contains supplementary material available at 10.1038/s41598-026-50360-1.

## Introduction

Colorectal cancer (CRC) remains a significant global health challenge and a leading cause of cancer-related mortality^1^. Among prognostic tools, the TNM (Tumor, Node, Metastasis) classification system, updated to its 8th edition by the American Joint Committee on Cancer (AJCC), is widely used for clinical staging and outcome prediction^2–4^. Metastasis to lymph nodes is a key mechanism in the process of cancer spread^5,6^. In the AJCC 8th edition TNM classification for colon cancer (CC), the N category is defined by the number of identified metastatic lymph nodes (MLN)^7^. While effective for risk stratification, the TNM system does not incorporate several potentially relevant aspects of lymph node assessment, including variability in the total number of examined lymph nodes.

LNR, defined as the ratio of pathologically positive lymph nodes to the total number of examined nodes, has been investigated as a prognostic marker in various malignancies, including colorectal cancer^8–16^. In colorectal cancer, a higher LNR has frequently been associated with worse survival outcomes, suggesting that LNR may capture nodal tumour burden in relation to nodal yield^17^. The investigation into the relationship between LNR and survival in stage III colon cancer patients was initially undertaken by Wang J. et al. using data from the Surveillance, Epidemiology, and End Results (SEER) cancer registry^17^. That analysis suggested an association between higher LNR and poorer survival among node-positive patients. Furthermore, the prognostic significance of LNR is particularly notable when fewer than 12 lymph nodes are examined, emphasizing the need for adequate lymph node assessment during surgery^17^. Ceelen et al., in a systematic review and meta-analysis of studies in stage III colorectal cancer, reported LNR as a prognostic factor for overall survival (OS) and disease-free survival (DFS), while also highlighting substantial heterogeneity and the predominantly retrospective design of included studies^18^. Overall, the existing evidence base remains largely retrospective, and prospective multicentre data are limited.

Several studies have discussed the relationship between the prognostic role of LNR and the adequacy of lymph node evaluation^19–23^. Current guidelines also specify a minimum of 12 lymph nodes in colon cancer surgery to minimize the false-negative rate of lymph node metastasis and to prevent under-staging^3,4,7^. A higher lymph node yield has been associated with improved long-term outcomes in some observational studies, although the underlying mechanisms are multifactorial and may reflect surgical technique, pathological assessment, tumour biology and host factors^24–28^. Given these considerations, the clinical interpretation of nodal metrics should acknowledge that the number of examined lymph nodes is not solely a biological variable but can also be influenced by perioperative and pathological processes.

In recent years, other nodal metrics, such as the log odds of positive lymph nodes (LODDS), have also been explored as prognostic indicators^34,35,37–39^. However, LODDS is closely related mathematically to LNR, and available evidence suggests broadly comparable prognostic performance in colon cancer, while LNR retains the advantage of simplicity and ease of calculation. Accordingly, LNR may be considered a pragmatic measure of nodal involvement, although its role relative to conventional nodal staging requires careful interpretation.

The aim of this prospective multicentre cohort study was to evaluate the association between LNR and overall survival in patients with stage I–III colon cancer, accounting for selected clinical and surgically relevant factors. A crucial aspect of our analysis was the use of a multivariable Cox proportional hazards model to examine the relationship between OS and LNR while incorporating covariates relevant to both oncological risk and perioperative course, including body mass index (BMI) and the severity of postoperative complications classified using the Clavien–Dindo scale.

Our research is focusing on patients diagnosed with CC (stage I-III). Although colon and rectal cancers are commonly grouped under CRC, the surgical and oncological management as well as the tumor biology differ between these locations^43,44^. Restricting the cohort to colon cancer was intended to reduce clinical heterogeneity and to support a more clinically coherent interpretation of the association between LNR and survival.

## Materials and methods

### Study design and population

This prospective multicentre cohort study was conducted from January 1, 2017 to June 30, 2018 across seven Polish surgical centers. Patients were followed for survival outcomes until September 30, 2022. The study enrolled adult patients aged 18–75 years with histologically confirmed primary CC, including sigmoid-rectal junction cancer (ICD-10 codes C18.0–C19), who were scheduled for elective radical surgical resection with curative intent and without clinical or radiological evidence of distant metastases at the time of surgery.

Exclusion criteria included age below 18 or above 75 years, refusal of surgical treatment, history of another malignancy within the preceding five years, and prior radiotherapy, chemotherapy, or immunotherapy for colorectal cancer. Patients undergoing palliative procedures were not included.

All study procedures were conducted in accordance with relevant guidelines and regulations. The study protocol was approved by the Bioethics Committee of the Medical University of Gdańsk (approval number NKBBN/237/2016). Written informed consent was obtained from all participants prior to inclusion.

### Preoperative assessments

Baseline data were collected on the day of hospital admission prior to surgery. Recorded variables included age, gender, body mass index (BMI), and preoperative nutritional risk assessed using the Nutritional Risk Screening 2002 (NRS2002) tool^46^. Age was recorded in full years. BMI was calculated as weight in kilograms divided by height in metres squared. The NRS-2002 score ranges from 0 to 7, with scores ≥ 3 indicating increased nutritional risk. Preoperative nutritional status was recorded as part of baseline clinical assessment but was not included in the primary multivariable model.

### Histopathological evaluation

Histopathological assessment was performed according to the AJCC 8th edition TNM classification^2^. Evaluated variables included tumour stage, histological grade, presence of lymphovascular invasion (LVI), total number of examined lymph nodes, and number of metastatic lymph nodes. The lymph node ratio (LNR) was calculated as the number of metastatic lymph nodes divided by the total number of examined lymph nodes.

### Surgical complications

Postoperative complications were prospectively recorded and classified using the Clavien-Dindo classification system^48,49^. For the purposes of analysis, complications were dichotomized into none or minor complications (Clavien-Dindo grades I-II) and severe complications (grades III-V). This categorization was chosen to reflect clinically relevant events requiring interventional management and to allow robust modelling of postoperative risk.

### Follow up and study endpoints

Postoperative follow-up was conducted in accordance with European Society for Medical Oncology (ESMO) guidelines for colon cancer^4^. The primary endpoint of the study was overall survival (OS), defined as time from surgical resection to death from any cause. Survival status was ascertained using the national Universal Electronic System for Registration of the Population (PESEL), ensuring complete follow-up for mortality regardless of changes in residence or treating institution.

Disease-free survival (DFS) was not analysed, as recurrence data were not collected in a standardized manner across all participating centers. This limitation is acknowledged and addressed in the Discussion section. Decisions regarding the use of adjuvant chemotherapy were made locally at each centre in accordance with national and ESMO guidelines; detailed data on adjuvant treatment were not uniformly available for analysis.

### Statistical analysis

Missing data were addressed using multiple imputation with chained equations, generating 40 imputed datasets and implemented using the mice package in R^50,51^.

The association between LNR and overall survival was examined using a multivariable Cox proportional hazards regression model. Covariates included age, sex, BMI, LVI, histological grade, disease stage (AJCC I–III), and the presence of severe postoperative complications (Clavien-Dindo grade ≥ III). These variables were selected a priori based on clinical relevance and prior literature, rather than data-driven selection^17–23,48,49,52^.

Kaplan–Meier survival curves were generated to visualize survival probabilities across selected subgroups. The number of patients at risk and 95% confidence intervals were reported. All analyses were performed using R software version 4.3.1. A two-sided p-value ≤ 0.05 was considered statistically significant.

### Sample size calculations

Sample size was calculated based on the number of events required for a multivariable Cox proportional hazards model with a continuous predictor (LNR), using the Schoenfeld method with the extension for non-binary covariates described by Hsieh and Lavori^63,64^. We assumed a two-sided α = 0.05, 80% power, a hazard ratio of 2.36 for LNR, its standard deviation of 0.3 derived from previously published data, and a coefficient of multiple correlation with other model covariates (R^2^) of 0.2^65^. Given these assumptions, 148 events were required. With an expected event rate of 35%, the target sample size equaled 423 participants.

## Results

### Baseline characteristics

A total of 423 patients were included in the final analysis (Table 1). The mean age of the study population was 62.42 ± 9.21 years, and 210 patients (49.6%) were female. The mean BMI was 27.97 ± 5.17 kg/m^2^. Of the 445 patients initially enrolled, 22 were excluded from the final analysis following withdrawal of consent for further contact and processing of personal data, resulting in a study cohort of 423 patients.

**Table 1:** Baseline characteristics of the study cohort (*N* = 423).

Characteristics	Overall (*N* = 423)
Age (mean ± SD)	62.42 (9.21)
Sex (%)	
Female	210 (49.6)
Male	213 (50.4)
Lymph nodes ratio (mean ± SD)	0.07 (0.14)
Tumor histological grade (%)	
G1	41 (10.0)
G2	318 (77.8)
G3	50 (12.2)
Tumor stage (%)	
I	74 (17.5)
II	189 (44.7)
III	160 (37.8)
NRS2002 score	
Less than 3	289 (72.1)
At least 3	112 (27.9)
BMI (mean ± SD)	27.97 (5.17)
Number of dissected lymph nodes (%)	
Less than 12	100 (24.1)
At least 12	315 (75.9)
Lymph node metastases (%)	
No	264 (63.8)
Yes	150 (36.2)
Lymphovascular invasion (%)	
No	296 (70.6)
Yes	123 (29.4)
Surgical complications (Clavien-Dindo grade) (%)	
Less than grade III or no complications	390 (92.9)
At least grade III	30 (7.1)

alues are presented as mean ± SD or n (%). BMI, body mass index; LNR, lymph node ratio; LVI, lymphovascular invasion; NRS 2002, Nutritional Risk Screening 2002; SD, standard deviation.

Regarding lymph node assessment, 100 patients (24.1%) had fewer than 12 lymph nodes examined, while 315 patients (75.9%) had 12 or more lymph nodes retrieved. Lymph node metastases were identified in 150 patients (36.2%), whereas 264 patients (63.8%) had no metastatic lymph nodes. LVI was present in 123 patients (29.4%) and absent in 296 patients (70.6%).

Based on postoperative histopathological evaluation according to the AJCC 8th edition classification, 74 patients (17.5%) were classified as stage I, 189 patients (44.7%) as stage II, and 160 patients (37.8%) as stage III. Tumour grading revealed 41 patients (10.0%) with grade G1, 318 patients (77.8%) with grade G2, and 50 patients (12.2%) with grade G3 disease.

Postoperative complications occurred in 70 patients (16.0%). Minor complications (Clavien-Dindo grades I-II) were observed in 36 patients (8.6%), while severe complications (grades III-V) occurred in 30 patients (7.1%). Three deaths occurred within 30 days after surgery.

Eleven observations had missing values for the lymph node ratio and were handled using multiple imputation, as described in the Statistical Methods section.

### Follow-up and survival

The mean duration of follow-up for the entire cohort, including both deaths and censored observations, was 1751 days, while the median follow-up time was 1858 days, with an interquartile range (IQR) of 1741–1971 days.

Among censored observations, the mean follow-up duration was 1889 days and the median follow-up duration was 1894 days, with an IQR of 1797–1998 days.

### Multivariable cox regression analysis

Results of the multivariable Cox proportional hazards regression analysis are presented in Table 2. After adjustment for age, sex, disease stage, tumour grade, LNR, BMI, LVI, and postoperative complications, LNR, BMI, and the presence of severe postoperative complications (Clavien-Dindo grade ≥ III) were independently associated with OS.

**Table 2:** Multivariable Cox proportional hazards model for overall survival in patients with colon cancer.

Characteristic	HR	95% CI	*p*-value
Age	1.02	0.98, 1.05	0.3
Sex			
Male	–	–	
Female	1.23	0.74, 2.06	0.4
BMI	1.07	1.02, 1.13	**0.007**
Grade III Clavien-Dindo or more			
No	–	–	
Yes	2.25	1.06, 4.78	**0.035**
Lymphovascular invasion			
No	–	–	
Yes	1.53	0.89, 2.61	0.12
LNR (per 0.1 unit)	1.26	1.07, 1.48	**0.006**
Stage			
I	–	–	
II	1.11	0.46, 2.68	0.8
III	1.02	0.39, 2.67	> 0.9
Grading			
G1	–	–	
G2	1.03	0.35, 3.03	> 0.9
G3	2.12	0.64, 7.02	0.2

Hazard ratios (HRs) are shown with 95% confidence intervals (CIs). BMI, body mass index; CI, confidence interval; HR, hazard ratio; LNR, lymph node ratio; LVI, lymphovascular invasion.

For LNR, each 0.1-unit increase was associated with a 26% increase in the hazard of death (hazard ratio [HR] 1.26, 95% confidence interval [CI] 1.07–1.48, *p* = 0.006), holding all other variables in the model constant.

BMI was also associated with overall survival. Each one-unit increase in BMI was associated with a 7% increase in the hazard of death (HR 1.07, 95% CI 1.02–1.13, *p* = 0.007).

Patients who experienced severe postoperative complications had a significantly higher risk of death compared with those without such complications. The presence of complications classified as Clavien-Dindo grade ≥ III was associated with a 2.25-fold increase in the hazard of death (HR 2.25, 95% CI 1.06–4.78, *p* = 0.035).

After adjustment for covariates included in the model, LVI was not significantly associated with overall survival (HR 1.53, 95% CI 0.89–2.61, *p* = 0.12). Similarly, disease stage was not significantly associated with overall survival in the multivariable model, with no statistically significant differences observed for stage II or stage III compared with stage I.

### Kaplan-meier survival analyses

**Fig. 1:**
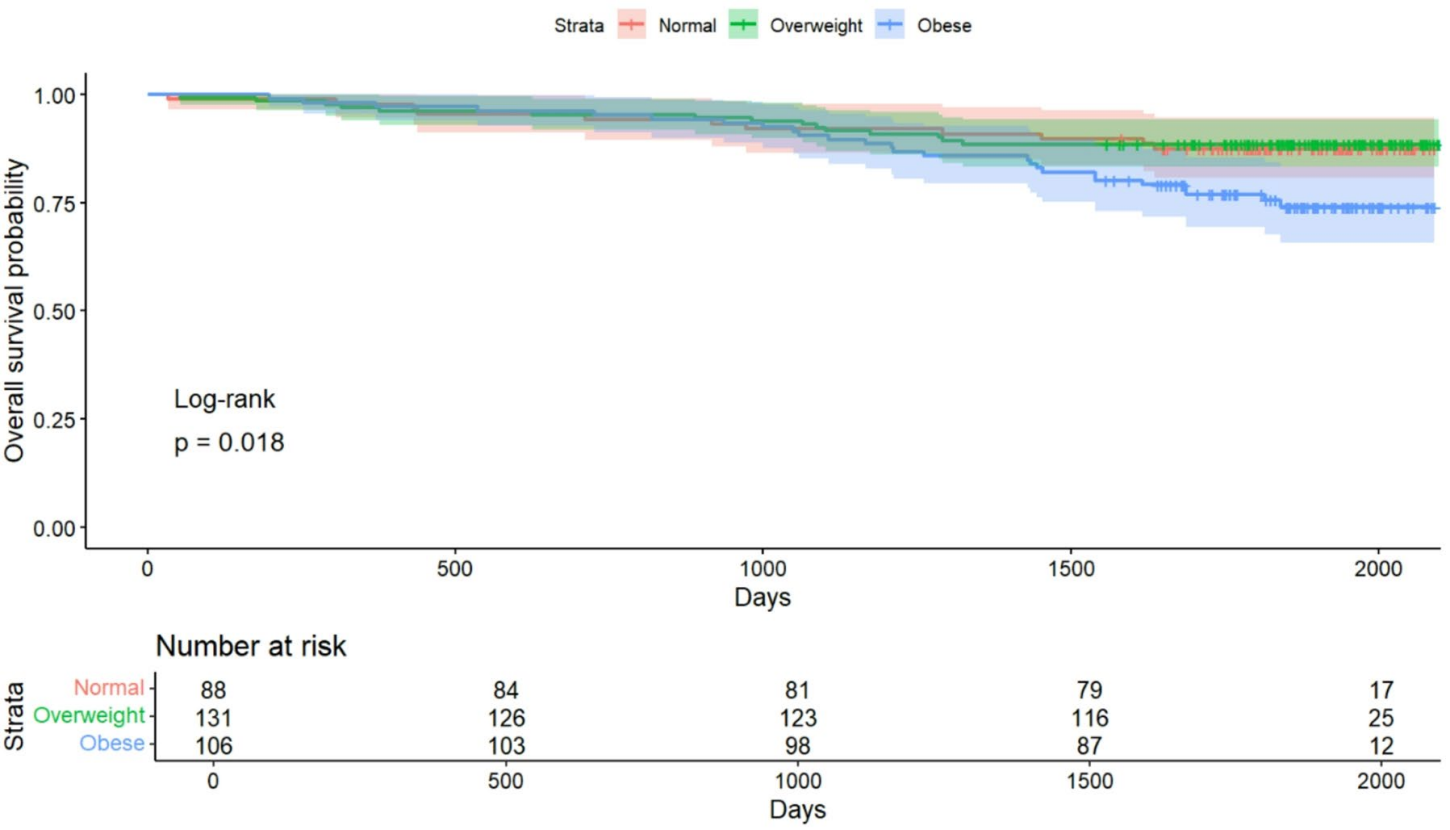
Kaplan-Meier curves for overall survival stratified by BMI categories. Curves are shown for normal weight, overweight, and obese patients; six underweight patients were excluded. Point estimates with 95% confidence intervals are presented, and the number of patients at risk is shown below the plot.

**Fig. 2:**
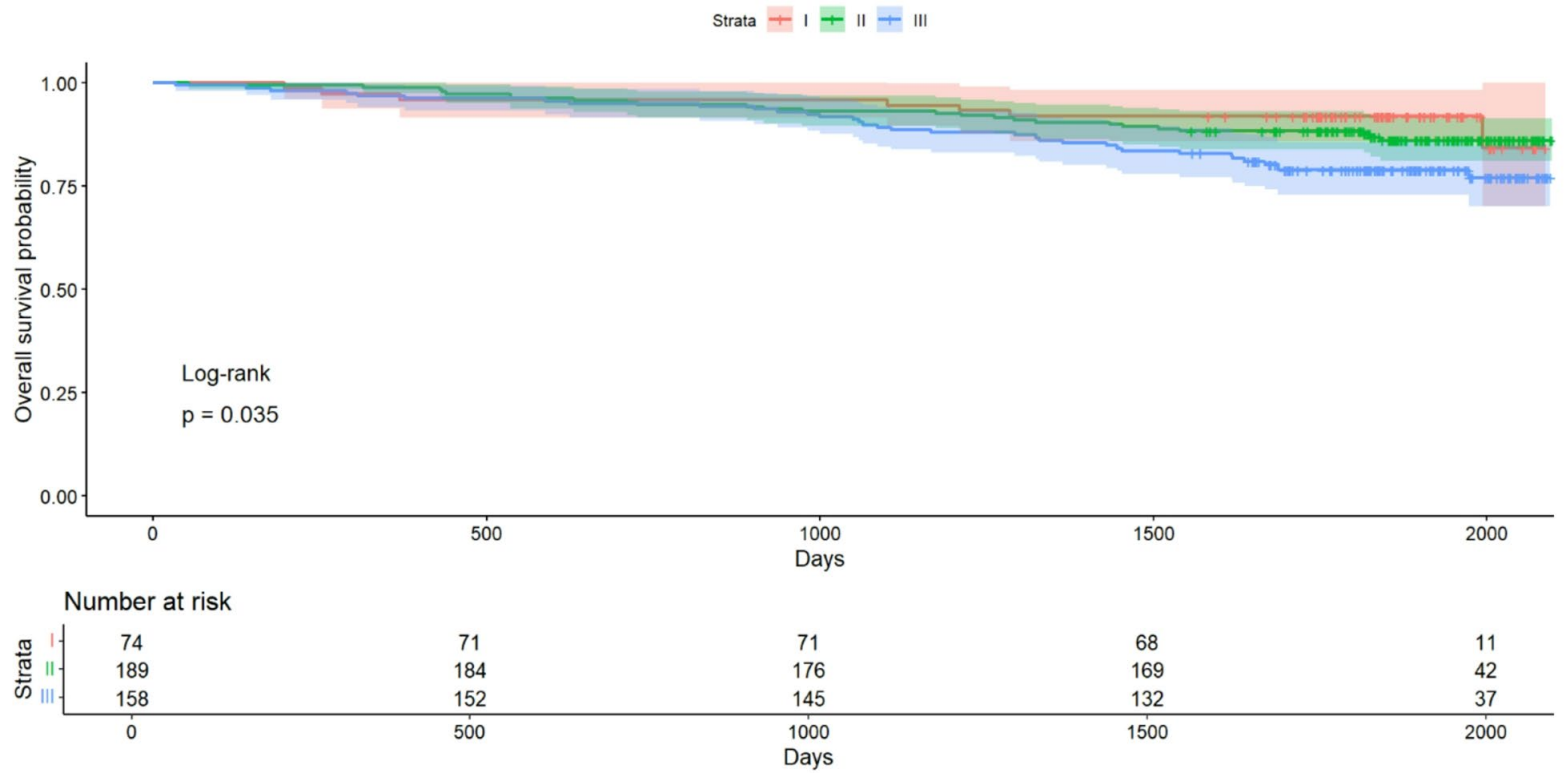
Kaplan-Meier curves for overall survival stratified by AJCC stage (I-III). Point estimates with 95% confidence intervals are presented, and the number of patients at risk is shown below the plot.

Kaplan-Meier survival curves for overall survival stratified by BMI categories are shown in Fig. 1. Kaplan-Meier curves stratified by disease stage (I-III) are presented in Fig. 2.

The corresponding Kaplan-Meier survival estimates with 95% confidence intervals at selected time points are provided in the Supplementary Material (Supplementary Tables S1 and S2).

The numbers of patients at risk and the corresponding 95% confidence intervals for survival estimates are provided in the tables accompanying each figure.

### Exploratory subgroup analyses according to lymph node yield and disease stage

As exploratory subgroup analyses, multivariable Cox proportional hazards models with the same covariate structure as in the primary analysis were fitted separately for patients with different lymph node yield (< 12 vs. ≥12 examined lymph nodes). In patients with ≥ 12 examined lymph nodes, lymph node ratio (LNR) remained statistically significantly associated with overall survival, with effect estimates comparable in direction to those observed in the primary model (Supplementary Table S3). In contrast, in patients with fewer than 12 examined lymph nodes, the association between LNR and overall survival did not reach statistical significance, although the direction of the effect estimate was consistent with the primary analysis and was accompanied by wide confidence intervals, suggesting limited statistical power in this subgroup (Supplementary Table S4).

To further explore the association between LNR and overall survival in a clinically relevant subgroup, an additional exploratory analysis was performed in patients with stage III disease. In this subgroup, LNR remained significantly associated with overall survival after adjustment for clinical and surgical covariates (Supplementary Table S5). Across all subgroup analyses, the direction of effect estimates for other covariates, including body mass index and severe postoperative complications (Clavien–Dindo grade ≥ III), was consistent with the primary multivariable model.

## Discussion

In this prospective multicentre cohort study, a significant association between LNR and OS was observed in patients with CC. A 0.1-unit increase in LNR was associated with a 26% higher hazard of death during the observation period. In exploratory subgroup analyses stratified by lymph node yield, the association between lymph node ratio and overall survival was statistically significant in patients with ≥ 12 examined lymph nodes. In contrast, among patients with fewer than 12 examined lymph nodes, effect estimates for LNR were directionally consistent with the primary analysis but did not reach statistical significance and were accompanied by wide confidence intervals, suggesting limited statistical power in this subgroup. These findings indicate that the prognostic performance of LNR may be influenced by the adequacy of lymph node evaluation. These findings are consistent with previous studies reporting an association between LNR and survival outcomes in CC and CRC^17–19,23^.

Several researchers have attempted to incorporate the LNR into the traditional staging system of CRC, focusing on the determination of specific cutoff points to enhance prognostic accuracy. Berger et al. demonstrated that LNR cutoffs below 5%, 5% to 20%, 20% to 40%, and above 40% significantly predicted DFS and OS in stage II and III CC patients treated with adjuvant therapy^19^. Wang et al. proposed categorising LNR into four subgroups based on predefined ratio ranges (< 1/14, 1/14 to < 0.25, 0.25 to < 0.50, and 0.50 to 1.0)^17^. Sabbagh et al. identified an LNR threshold of 10% as optimal for differentiating prognostic subgroups among stage III CC patients^23^. Moreover, Shinto et al. suggested that prognostically relevant LNR cutoffs may vary according to tumour location in stage III CC, proposing threshold values of 0.16 for right-sided and 0.22 for left-sided tumours^53^.

In the present cohort, fewer than 12 lymph nodes were examined in approximately 25% of patients, a proportion comparable to that reported in other multicentre and population-based studies^18,24^. Current clinical guidelines recommend the evaluation of at least 12 lymph nodes to reduce the risk of understaging in colon cancer; however, lymph node yield may be influenced by multiple factors beyond surgical technique, including tumour biology, patient-related characteristics and pathological assessment^3,4,7^. When analyses were stratified by lymph node yield (< 12 vs. ≥ 12 examined lymph nodes), the direction of the association between lymph node ratio and overall survival was broadly consistent across subgroups. However, statistical significance was observed only in patients with adequate lymph node evaluation (≥ 12 nodes), highlighting the importance of sufficient nodal yield for reliable prognostic assessment^18,22^.

A notable limitation of this study is the lack of detailed data on adjuvant therapies, which precluded adjustment for the potential effects of systemic treatment on survival outcomes. This limitation is particularly relevant given that most patients with stage III disease, as well as those classified as high risk in stage II, are candidates for adjuvant therapy^4^. Accordingly, the absence of adjuvant treatment data limits the interpretation of OS and should be considered when contextualising the present findings. This underscores the need for future studies to incorporate detailed treatment data to better delineate the interaction between nodal metrics and systemic therapy.

The present study did not include data on DFS. Most disease recurrences in CRC occur within the first years following surgical resection. In the COLOFOL trial, which included patients after high quality surgery for stage II and III CRC, 87.3% of recurrences were detected within the first three years after surgery, highlighting the importance of early postoperative surveillance^59^. From a surgical perspective, the radicality of resection, the extent of lymphadenectomy, and postoperative complications are critical determinants of long term prognosis. In the present cohort, severe postoperative complications (Clavien-Dindo grade ≥ III) occurred in 30 patients (7.1%) and were associated with a reduced probability of long term survival (HR 2.25, 95% CI 1.06–4.78). Perioperative deaths within 30 days after surgery (*n* = 3) were included in the survival analysis. Duraes et al. reported that postoperative complications negatively affect long term oncological outcomes, with increasing severity corresponding to poorer prognosis^60^. Similarly, Aoyama et al. demonstrated that postoperative complications adversely influence both OS and DFS, irrespective of adjuvant therapy or tumour location^61^. The incidence of severe postoperative complications observed in the present study is consistent with previously published data, including the report by Matsuda et al., who observed a Clavien-Dindo grade III or higher complication rate of 8.1%^62^. These findings support the inclusion of postoperative morbidity as a clinically relevant variable in prognostic models.

In this prospective multicentre cohort study, using multiple imputation and a multivariable Cox regression model, an association between LNR and OS was identified in patients with CC. The application of multiple imputation addressed missing data and reduced the risk of bias, which would be caused by dropping incomplete observations. The observed association between LNR, BMI, postoperative complications, and OS highlights the multifactorial nature of prognosis in CC. However, the present study was not designed to assess the incremental prognostic value of LNR over established nodal staging systems, and therefore no conclusions regarding its superiority or role in future staging classifications can be drawn. Although several authors have proposed alternative staging approaches incorporating lymph node ratio, these concepts require further validation and were beyond the scope of the present study^45^.

## Supplementary Information

Below is the link to the electronic supplementary material.

Supplementary Material 1

## Data Availability

The data supporting the findings of this study were collected and analyzed by the authors. These data are not publicly available but can be provided upon reasonable request. Requests for data access should be directed to the corresponding author at eq@gumed.edu.pl.
