# Supplementary material for: Lymph node ratio and overall survival in patients with colon cancer: a prospective multicentre cohort study
Source: Sci Rep. 2026 Apr 27;16:19290. doi: 10.1038/s41598-026-50360-1 (PMC13284318; doi:10.1038/s41598-026-50360-1)
Supplement: Supplementary file 1 — Supplementary Material 1 [file 41598_2026_50360_MOESM1_ESM.docx]

**Supplementary Table S1**. Kaplan-Meier overall survival estimates by BMI category. Kaplan-Meier survival probabilities with 95% confidence intervals at selected time points (500, 1000, 1500, and 2000 days) are provided for normal weight, overweight, and obese patients.

| Time [days] | | 500 | 1000 | 1500 | 2000 |
| --- | --- | --- | --- | --- | --- |
| KM survival estimate (95% CI) | Normal | 0.96 (0.93-1.00) | 0.92 (0.87-0.98) | 0.90 (0.84-0.96) | 0.87 (0.81-0.95) |
|  | Overweight | 0.96 (0.93-1.00) | 0.94 (0.90-0.98) | 0.89 (0.83-0.94) | 0.89 (0.83-0.94) |
|  | Obese | 0.97 (0.94-1.00) | 0.92 (0.88-0.98) | 0.82 (0.75-0.90) | 0.74 (0.66-0.83) |

**Supplementary Table S2.** Kaplan-Meier overall survival estimates by disease stage. Kaplan-Meier survival probabilities with 95% confidence intervals at selected time points (500, 1000, 1500, and 2000 days) are provided for AJCC stage I, II, and III patients.

| Time [days] | | 500 | 1000 | 1500 | 2000 |
| --- | --- | --- | --- | --- | --- |
| KM survival estimate (95% CI) | Stage I | 0.96 (0.92-1.00) | 0.96 (0.92-1.00) | 0.92 (0.86-0.98) | 0.84 (0.70-1.00) |
|  | Stage II | 0.97 (0.95-1.00) | 0.93 (0.90-0.97) | 0.89 (0.85-0.94) | 0.86 (0.81-0.91) |
|  | Stage III | 0.96 (0.93-0.99) | 0.92 (0.88-0.96) | 0.84 (0.78-0.90) | 0.77 (0.70-0.85) |

**Supplementary Table S3**. Multivariable Cox proportional hazards model for overall survival in patients with ≥12 examined lymph nodes. Hazard ratios (HRs) are shown with 95% confidence intervals (CIs). Covariates included age, sex, body mass index, tumour stage, histological grade, lymphovascular invasion, lymph node ratio, and severe postoperative complications (Clavien–Dindo grade ≥III).

| **Characteristic** | **HR** | **95% CI** | ***p*-value** |
| --- | --- | --- | --- |
| **Age** | 1.01 | 0.97, 1.05 | 0.5 |
| **Sex** |  |  |  |
| Male | — | — |  |
| Female | 1.25 | 0.67, 2.34 | 0.5 |
| **BMI** | 1.08 | 1.01, 1.15 | **0.029** |
| **Grade III Clavien-Dindo or more** |  |  |  |
| No | — | — |  |
| Yes | 1.22 | 0.35, 4.27 | 0.7 |
| **Lymphovascular invasion** |  |  |  |
| No | — | — |  |
| Yes | 1.44 | 0.76, 2.74 | 0.3 |
| **LNR** (per 0.1 unit) | 12.0 | 1.59, 90.4 | **0.018** |
| **Stage** |  |  |  |
| I | — | — |  |
| II | 1.02 | 0.30, 3.44 | >0.9 |
| III | 1.03 | 0.28, 3.76 | >0.9 |
| **Grading** |  |  |  |
| G1 | — | — |  |
| G2 | 0.84 | 0.23, 3.03 | 0.8 |
| G3 | 1.79 | 0.43, 7.52 | 0.4 |

**Supplementary Table S4**. Multivariable Cox proportional hazards model for overall survival in patients with <12 examined lymph nodes. Hazard ratios (HRs) are shown with 95% confidence intervals (CIs). Covariates included age, sex, body mass index, tumour stage, histological grade, lymphovascular invasion, lymph node ratio, and severe postoperative complications (Clavien–Dindo grade ≥III).

| **Characteristic** | **HR** | **95% CI** | ***p*-value** |
| --- | --- | --- | --- |
| **Age** | 1.03 | 0.95, 1.11 | 0.5 |
| **Sex** |  |  |  |
| Male | — | — |  |
| Female | 1.17 | 0.38, 3.63 | 0.8 |
| **BMI** | 1.08 | 0.97, 1.20 | 0.12 |
| **Grade III Clavien-Dindo or more** |  |  |  |
| No | — | — |  |
| Yes | 4.29 | 1.00, 18.5 | 0.050 |
| **Lymphovascular invasion** |  |  |  |
| No | — | — |  |
| Yes | 1.71 | 0.49, 6.03 | 0.4 |
| **LNR** (per 0.1 unit) | 4.20 | 0.08, 210 | 0.4 |
| **Stage** |  |  |  |
| I | — | — |  |
| II | 1.43 | 0.27, 7.68 | 0.6 |
| III | 0.90 | 0.11, 7.42 | >0.9 |
| **Grading** |  |  |  |
| G1 | — | — |  |
| G2 | 1.57 | 0.13, 18.7 | 0.7 |
| G3 | 4.01 | 0.25, 63.3 | 0.3 |

**Supplementary Table S5**. Multivariable Cox proportional hazards model for overall survival in patients with stage III colon cancer. Hazard ratios (HRs) are shown with 95% confidence intervals (CIs). Covariates included age, sex, body mass index, histological grade, lymphovascular invasion, lymph node ratio, and severe postoperative complications (Clavien–Dindo grade ≥III).

| **Characteristic** | **HR** | **95% CI** | ***p*-value** |
| --- | --- | --- | --- |
| **Age** | 0.98 | 0.94, 1.02 | 0.3 |
| **Sex** |  |  |  |
| Male | — | — |  |
| Female | 1.04 | 0.49, 2.25 | >0.9 |
| **BMI** | 1.11 | 1.03, 1.20 | **0.008** |
| **Grade III Clavien-Dindo or more** |  |  |  |
| No | — | — |  |
| Yes | 2.84 | 1.06, 7.61 | **0.039** |
| **Lymphovascular invasion** |  |  |  |
| No | — | — |  |
| Yes | 2.58 | 1.19, 5.58 | **0.018** |
| **LNR** (per 0.1 unit) | 10.4 | 1.73, 62.4 | **0.013** |
| **Grading** |  |  |  |
| G1 | — | — |  |
| G2 | 1.05 | 0.12, 9.15 | >0.9 |
| G3 | 1.31 | 0.14, 12.2 | 0.8 |
